# Effectiveness of Virtual Reality on Postoperative Pain, Disability and Range of Movement after Knee Replacement: A Systematic Review and Meta-Analysis

**DOI:** 10.3390/life14030289

**Published:** 2024-02-21

**Authors:** Jara Esteban-Sopeña, Hector Beltran-Alacreu, Marc Terradas-Monllor, Juan Avendaño-Coy, Nuria García-Magro

**Affiliations:** 1 Facultad de Ciencias de la Salud, Universidad Francisco de Vitoria, Pozuelo de Alarcón, 28223 Madrid, Spain; jaramaria.esteban@ufv.es (J.E.-S.); nuria.garcia@ufv.es (N.G.-M.); 2 Hospital Universitario del Sureste, Arganda del Rey, 28500 Madrid, Spain; 3 Escuela Internacional de Doctorado, Universidad de Castilla-La Mancha, 13071 Ciudad Real, Spain; 4 Toledo Physiotherapy Research Group (GIFTO), Faculty of Physical Therapy and Nursing, Universidad de Castilla-La Mancha, 45071 Toledo, Spain; juan.avendano@uclm.es; 5 Research Group on Methodology, Methods, Models and Outcomes of Health and Social Sciences (M3O), Faculty of Health Sciences and Welfare, Centre for Health and Social Care Research (CESS), Universidad de Vic-Universidad Central de Cataluña (UVic-UCC), 08500 Vic, Spain; marc.terradas@uvic.cat; 6 Pain Medicine Section, Anaesthesiology Department, Hospital Clinic de Barcelona, 08036 Barcelona, Spain

**Keywords:** pain, postoperative pain, total knee arthroplasty, virtual reality, augmented reality, systematic review, meta-analysis

## Abstract

Postoperative pain after knee arthroplasty (TKA) is a reality that continues to be experienced today. Recently, virtual reality (VR) has demonstrated effectiveness in the management of pain. Our aim was to review the original controlled trials evaluating the effectiveness of VR for pain management and quality of life after TKA. Six databases were searched for articles published from inception to September 2023, following (PRISMA) guidelines. The methodological quality was assessed using the Risk of Bias tool for Randomized Trials (ROB2). Five RCTs were included in the systematic review, and four of them in the meta-analysis. The effectiveness of VR for short term pain relief was superior compared to the control (MD = −0.8 cm; CI 95%: −1.3 to −0.4; *p* < 0.001). VR showed a greater effect on the secondary outcomes of WOMAC (MD = −4.6 points; CI 95%: −6.5 to −2.6, *p* < 0.001) and the HSS scale (MD = 6.5 points; CI 95%: 0.04 to 13.0, *p* = 0.049). However, no differences were found in the effect on the ROM between groups (MD = 3.4 grades; CI 95%: −6.0 to 12.8, *p* = 0.48). Our findings suggest that the use of virtual reality during the postoperative period could be an effective non-pharmacological therapy in relieving acute pain, compared to a control intervention, with a very low degree of certainty according to the Grades of Recommendation, Assessment, Development, and Evaluation (GRADE). However, the low methodological quality of the articles limits our findings.

## 1. Introduction

Knee osteoarthritis is the most prevalent articular chronic disease, and its incidence increases yearly mainly due to the ageing of the population, sedentary lifestyles, obesity, and increased life expectancy [1]. Among the different treatments, knee arthroplasty is an effective treatment for symptomatic, end-stage knee osteoarthritis [2]. This surgical procedure has been shown to improve osteoarthritis patients’ quality of life by providing significant pain relief and improving their functional capacity [2,3,4]. Therefore, knee arthroplasty has become one of the most popular surgical procedures, and the number of arthroplasties has increased in developed countries [2,5]. Specifically in Spain, the annual estimate of surgeries was a progression from 12,500 interventions in 1995 to 25,000 interventions in 2000 and around 42,400 interventions in 2008 [5,6]. Moreover, compound annual growth of 11.5% was observed between 1997 and 2008 [5].

Despite being a successful intervention, severe postoperative acute pain after a knee arthroplasty remains a widespread but still underestimated problem [7]. A study comparing pain intensities after 179 different surgical procedures in 50,523 patients concluded that knee arthroplasty was one of the surgeries with the highest acute pain ratings [7]. Moreover, acute postoperative pain after a knee arthroplasty is not only intense but moderately prevalent. A recent study showed that 51% of patients who received a total knee arthroplasty (TKA) reported moderate to severe acute postoperative pain (>4 on a 10-point numeric rating scale), which is higher than that reported in other orthopedic surgeries, such as total hip arthroplasty [8].

Despite advances in the pathophysiological mechanisms underlying acute pain, the development of new drugs and the application of multimodal techniques for its treatment, postoperative pain remains an unsolved problem [9]. The latest clinical guidelines for the management of acute postoperative pain from the American Pain Society (APS) recommend “the use of multimodal analgesia” [9], understood as “the combination of two or more drugs and/or analgesic methods, in order to enhance analgesia and reduce side effects” [10], for example, the combination of NSAIDs and regional analgesia techniques. However, despite the recommendations, the use of multimodal analgesia in Spain is not frequent [11]. 

Taking into account the recommendations of the APS, virtual reality for pain management, as a non-pharmacological measure, in combination with pharmacological methods, is offered as a promising therapy. Virtual reality (VR) is an emerging and innovative therapeutic measure showing great potential for pain relief in different medical contexts, whether during the postoperative period, chronic disease, or certain painful procedures [12]. This tool provides patients with a non-invasive and non-pharmacological approach, with reduced adverse effects, to manage pain and improve quality of life. In addition, it could contribute to reducing health care costs by decreasing the use of analgesics and opioids during the postoperative period and consequently decreasing side effects during recovery.

To our knowledge, no meta-analysis has been carried out to critically evaluate the intervention effects of VR on acute postoperative pain and quality of life after total knee arthroplasty. Therefore, we aimed to conduct a meta-analysis of controlled trials through multiple literature searches to investigate the potential effectiveness of VR in reducing postoperative pain and improving quality of life in patients undergoing total knee arthroplasty. The hypothesis was that VR therapy would result in a postoperative pain reduction and improved quality of life after TKA.

## 2. Materials and Methods

The present systematic review and meta-analysis was conducted in accordance with the Preferred Reporting Items for Systematic Reviews and Meta-Analyses (PRISMA) guidelines [13] and has been registered in the PROSPERO International Prospective Register of Systematic Reviews (reference number: CRD42023442572). 

### 2.1. Data Sources and Searches

A search for randomized controlled trials (RCTs) was conducted without temporal restrictions, concluding on 30 June 2023. The PubMed, Cochrane, Lilacs, CINAHL, Medline, and Scopus databases were searched. Two reviewers (N.G.M. and J.E.S.) independently carried out the search to minimize bias, adhering to a mutually agreed-upon methodology for the formulation of search equations. In cases of disagreement, a third researcher (H.B.A.) intervened to achieve consensus. The search equations employed were formulated through the combination of the following MeSH terms and keywords: “arthroplasty replacement”, “knee replacement”, “total knee arthroplasty”, “TKA”, “data display”, “virtual reality”, “VR”, “virtual reality therapy”, “augmented reality”, “pain”, “acute pain”, “pain management”, “pain relief”, “analgesia”, “quality of life”, and “QoL”. The specific search string was - (“virtual reality”[MeSH Terms] OR (“virtual”[All Fields] AND “reality”[All Fields]) OR “virtual reality”[All Fields] OR (“vis resour”[Journal] OR “proc ieee virtual real conf”[Journal] OR “vr”[All Fields])) AND (“arthroplasty, replacement, knee”[MeSH Terms] OR “TKA”[All Fields]) AND (“pain”[MeSH Terms] OR “pain”[All Fields] OR (“pain management”[MeSH Terms] OR (“pain”[All Fields] AND “management”[All Fields]) OR “pain management”[All Fields])).

### 2.2. Study Selection

Study selection adhered to the criteria outlined in the PRISMA checklist’s PICO(S) framework (P—Participants: adult (+45 years old) patients who had undergone surgical knee replacement ; I—Interventions: utilization of virtual reality in any modality (passive, exploratory or interactive) for pain postoperative reduction; C—Comparator: the presence of a comparator (e.g., placebo, standard therapy, no treatment, gold standard, inactive controls, active controls, etc.) was not a requisite for study inclusion; O—Outcomes: self-rated pain and quality of life assessed through patient-reported outcome measures, specifically the Visual Analogue Scale and Numeric Pain Scale for pain, WOMAC and EQ-5D for functionality and quality of life and Range of Movement (ROM); S—Study Design: randomized clinical trials). Moreover, studies were included if the published language was either English or Spanish. Exclusion criteria were as follows: systematic reviews, meta-analyses, opinion pieces, publications in abstract form only, and duplicate articles. Methodologically, studies were also excluded if they included patients with chronic pain and/or prior neurological pathologies, and did not measure the effectiveness or effects of virtual reality. 

Two independent researchers (N.G.M. and J.E.S.) selected the studies based on these inclusion and exclusion criteria.

### 2.3. Data Extraction

Two authors (J.E.S. and N.G.M.) independently conducted a comprehensive literature review. In the initial screening phase, each reviewer excluded studies based on the study title and abstract information. If the title and abstract provided sufficient evidence for the study’s potential inclusion, the study advanced to the second screening phase. During this phase, studies that passed the initial screening were assessed in full-text form, and only those meeting all inclusion criteria were selected for analysis. Any discrepancies between the reviewers were resolved through a consensus process, moderated by a third reviewer (H.B.A.) after each phase.

### 2.4. Assessment of Risk of Bias 

The risk of bias was evaluated in accordance with guidelines from the Cochrane organization [14], utilizing the Risk-of-Bias Tool for Randomized Trials (RoB 2) [15]. Two independent reviewers (N.G.M. and J.E.S.) assessed the risk of bias, and any disagreements were resolved by a third investigator (H.B.A). The assessment encompassed five domains, each with several subdomains. Each domain was assigned a risk level, categorized into one of three tiers: “low”, “high”, or “some concerns”. Additionally, an overall risk of bias for each study was articulated. For a study to be classified as “low risk”, all domains had to be evaluated as low risk. Conversely, if a study had at least one domain assessed as high risk or one domain with some concerns, its overall risk was designated as “high risk” or “some concerns”, respectively. Furthermore, to assess publication bias for the primary variable, which was the self-reported pain intensity using the Visual Analog Scale (VAS Pain), a funnel plot and Egger’s test were examined.

### 2.5. Data Synthesis and Statistical Analysis

The inverse variance method was used to analyze the main outcome (VAS pain) and secondary outcomes (WOMAC, ROM, and Hospital for Special Surgery Knee-Rating Scale (HSS)). Statistical heterogeneity was evaluated using the chi-squared test (with statistical significance set at *p* < 0.05), and heterogeneity was measured by calculating the I^2^, with 25%, 50% and 75% representing low, moderate, and high heterogeneity, respectively [14]. Random-effect and fixed-effect analysis models were used when the heterogeneity I^2^ was greater or lower than 50%, respectively. The mean difference (MD) was obtained for all outcomes since they were expressed on the same scale in the included studies. Confidence intervals were set at 95% (CI 95%) for all variables. The data included in the analysis for outcome pain VAS were from a short-term follow-up; depending on the data reported in the studies, it ranged between 2 and 10 days after surgery. The data analyzed for the WOMAC outcome were at 6 months of follow-up; for ROM they were analyzed at 7 days after surgery; and they were analyzed at three months for the Hospital for Special Surgery Knee-Rating Scale (HSS) [16]. The Review Manager (RevMan) [Computer program]. Version 5.4. The Cochrane Collaboration, 2020, was used for the quantitative analysis. The quality of evidence was classified for each outcome as high, moderate, low, or very low following the Grades of Recommendation Assessment, Development, and Evaluation (GRADE) method [17,18]. A description of the certainty of evidence was presented in summary of findings tables using GRADEpro Guideline Development Tool Software (McMaster University and Evidence Prime, 2024). Available from https://www.gradepro.org/ (accessed on 20 December 2023). 

## 3. Results

### 3.1. Study Identification and Selection

**Figure 1:**
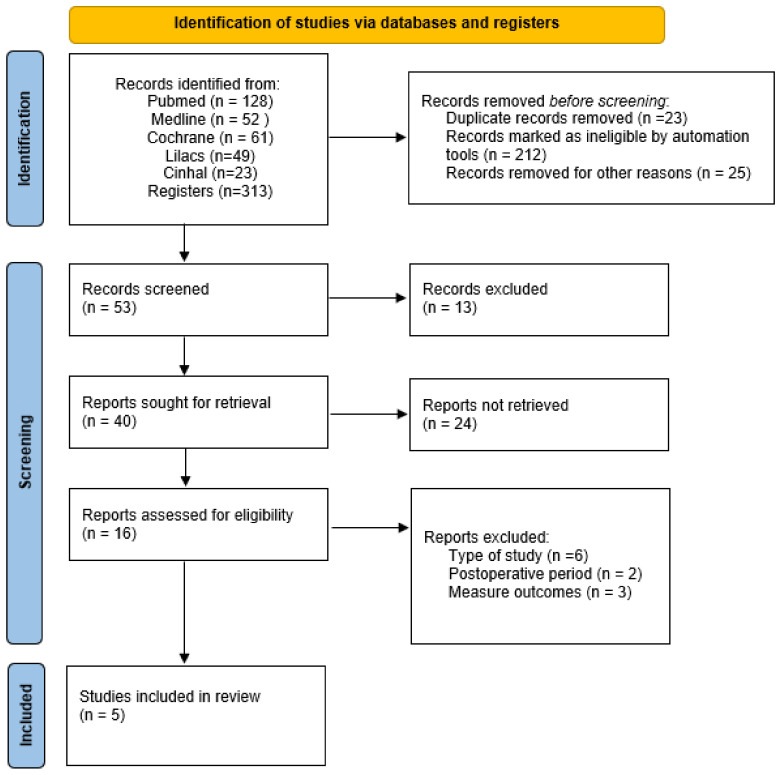
PRISMA flow diagram describing the screening process of the present systematic and meta-analysis review.

After screening 313 records based on the title and abstract, 53 full-text articles were selected for further evaluation. Upon full-text review, only five randomized controlled trials (RCTs) met the inclusion criteria for this systematic review, of which only four could be included in the meta-analysis. Figure 1 delineates the study selection process and the reasons for study exclusion.

### 3.2. Qualitative Summary of the Included Studies

All included studies compared the effect of VR in patients undergoing TKA. The characteristics of the included studies are summarized in Table 1.

#### 3.2.1. Study Characteristics

The publication dates ranged from 2018 to 2022, and the studies collectively enrolled a total of 264 participants. Detailed characteristics of the eligible studies are presented in Table 1.

All five randomized controlled trials (RCTs) included in the meta-analysis were published in English. These studies were conducted in various countries: China [19,23], Italy [20], the USA [21], and Israel [22]. The publication dates ranged from 2018 to 2022. Detailed characteristics of the eligible studies are presented in Table 1**.**

#### 3.2.2. Participant Characteristics

The systematic review incorporated five studies, comprising 264 participants for the qualitative analysis; the same number was included in the meta-analysis for quantitative evaluation. The sample sizes in the selected studies varied, ranging from 18 to 85 participants, with a mean of 51.5 participants per study. The mean age of participants spanned from 32 to 74 years, with a predominance of female patients. All five studies involved participants undergoing total knee arthroplasty (TKA), and one study [21] additionally included patients undergoing total hip arthroplasty (THA). 

#### 3.2.3. Interventions

The five studies compared VR with other interventions, e.g., traditional rehabilitation [19,20], conventional physiotherapy [22] and CPM equipment or spinal anesthesia [21]. All the interventions used an immersive VR device. The VR intervention was performed during surgery [21] and in the postoperative and rehabilitation period. The time duration ranged from 15 to 60 min [20,22,23]; Lingfeng did not specify the duration of the intervention [19]. As a traditional face-to-face care model, participants received VR therapy in a clinic [19,20,21,22,23].

Various VR programs were developed in the included studies.

In Lingfeng’s study [19], AR-based rehabilitation training technology was used. This technology mainly included three parts: real training data acquisition, virtual scene construction and virtual and real fusion. A model of the virtual scene was created from real images and parameters to guide the patient in his training. In Gianola et al.’s investigation, the patients performed passive knee motion on a Kinetect knee continuous passive motion system supported by a VR support system with games. They also carried out functional exercises (stair negotiation and level walking) daily for 60 min on at least 5 days [20]. In S. Barry et al’s. study, patients wore PICO G2 4K Enterprise VR glasses and Bose Quiet Comfort QC 35 noise-canceling headphones during surgery, including an anesthesia view and a side profile. Patients were able to choose among four different types of visual content (created by HypnoVR) and voice-guided sound/relaxation techniques [21]. In Lee Fuchs’ investigation, patients underwent, on postoperative days 1 and 2, daily physiotherapy sessions with a continuous passive motion (CPM) device and Samsung Gear VR glasses (a head-mounted display that allows the projection of a three-dimensional image). This physiotherapy session lasted 15 min. A nature film or a music film of the patient’s choice was projected onto the glasses before the intervention [22]. In the last study, the virtual reality intervention took place from the second day of the TKA. Patients were asked to row a boat in an immersive virtual environment. The intervention was performed three times a day for 30 min [23]. The characteristics and details of each intervention are listed in Table 1. In addition, a summary table of the virtual reality programs is included, organized in four dimensions: hardware, content (scenario), interaction and supervision (see Table 2).

#### 3.2.4. Outcome Measures

The main outcomes assessed in all included studies were pain and quality of life. Pain was measured using a VAS [19,20,21,22,23] and NRS [21]. These scales graded the pain intensity from 0 (no pain) to 10 (worst pain imaginable). Pain was also measured with the following indicators: quantity of sedation (preoperative and intraoperative), maximum heart rate, maximum systolic blood pressure, anesthesia time, OMEs, PACU sedative/narcotic usage and recovery duration [21]. In Gianola et al. study, the frequency of medication intake was also measured [20].

Disability was evaluated by the WOMAC scale in several articles [20,22,23]. This scale consists of 24 items measuring quality of life, in terms of symptomatology and physical disability, in people with osteoarthritis of the hip or knee [24]; it was also evaluated by the Health-related quality of life (HRQoL) tool and the EuroQol five-dimension questionnaire (EQ-5D) while the global perceived effect (GPE) was assessed by the GPE score [21]. The outcome measures are shown in Table 1.

#### 3.2.5. Risk of Bias within Included Studies

**Figure 2:**
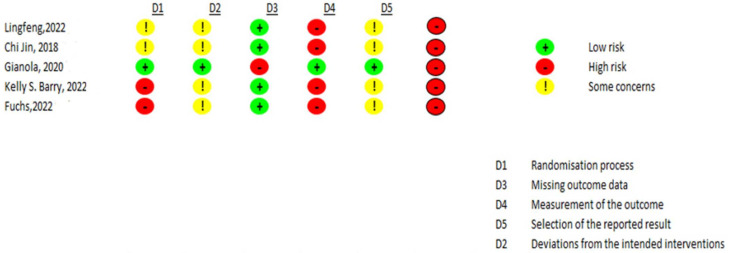
Risk of bias assessment within included studies using the ROB2 tool [19,20,21,22,23].

The researchers that assessed the risk of bias (N.G.M. and J.E.S.) agreed upon 75% of the items. Disagreements were mostly related to differentiating between an unclear and high risk of bias and were resolved by a third researcher (H.B.A). Figure 2 shows the risk of bias in the five included studies. The risk of selection bias was high in all the trials. Publication bias was observed for the primary variable, VAS pain (Egger´s test; t = −5.9, *p* = 0.027).

In the randomization process, the studies’ randomization method was not described even if they were RCTs [19,22,23], and, in article [21], the selection process was self-selected. Additionally, most of the trials did not report the method for allocation concealment, except one study [20]. Regarding missing result data, all studies presented a low risk, except one of them [20], which reported a loss of patients in the process that was not justified. In the measurement of results, almost all studies presented a high risk [19,21,22,23]. The results were only adequately reported in one study [20].

#### 3.2.6. Quantitative Summary of the Included Studies

**Figure 3:**
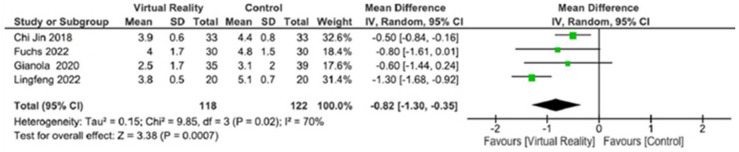
Forest plots from the meta-analysis represent the effect of virtual reality vs. controls on VAS scale. The green squares represent the mean difference for each study and the black diamond represents the aggregate average of the mean differences [19,20,22,23].

Four RCTs were included in the meta-analysis, with 240 participants: *n* = 118 participants in the experimental group and *n* = 126 in the control group. Figure 3 summarizes the trials that assessed the effect of the interventions on postoperative pain measured by VAS. The effectiveness of virtual reality for short-term pain relief (<10 days post-surgery) was superior compared to the control (MD = −0.8 cm; CI 95%: −1.3 to −0.4; *p* < 0.001), with moderate heterogeneity (I2 = 70%, *p* = 0.02). 

**Figure 4:**
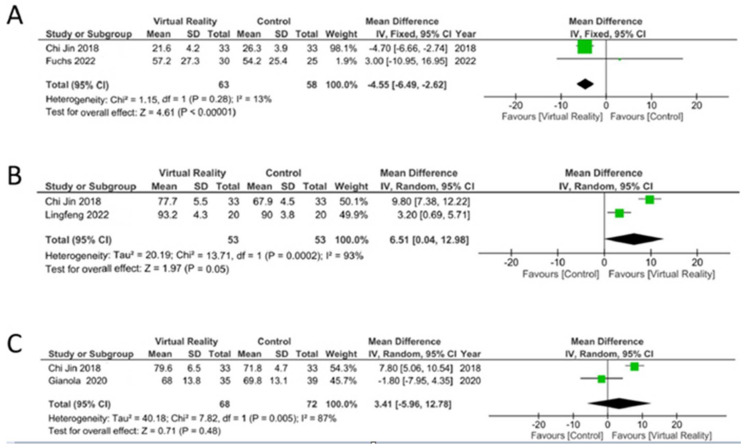
Forest plots from the meta-analysis represent the effect of virtual reality vs. controls on WOMAC (**A**), HSS (**B**) and ROM (**C**) outcomes in long, medium and short term, respectively. The green squares represent the mean difference for each study and the black diamond represents the aggregate average of the mean differences [19,20,22,23].

Figure 4 summarizes the trials that assessed secondary outcomes. The effectiveness of virtual reality for the WOMAC outcome at long-term follow-up (6 months post-surgery) was superior to that of the control (MD = −4.6 points; CI 95%: −6.5 to −2.6., *p* < 0.001), with low heterogeneity (I^2^ = 13%, *p* = 0.28) (Figure 4A). A greater effect of the virtual reality intervention was shown on the HSS outcome (MD = 6.5 points; CI 95%: 0.04 to 13.0, *p* = 0.049), with high heterogeneity (I^2^ = 93%, *p* < 0.001) in the medium term (3 months post-surgery) (Figure 4B). No differences were found between the experimental and control groups in the ROM outcome (MD = 3.4 grades; CI 95%: −6.0 to 12.8, *p* = 0.48), with high heterogeneity (I^2^ = 87%, *p* < 0.01) in the short term (7 days post-surgery) (Figure 4C).

The certainty of evidence according to GRADE was very low for the main outcome of pain VAS. For secondary outcomes, it was low for the WOMAC outcome and very low for the HSS and ROM (see Table 3).

## 4. Discussion

Our systematic review and meta-analysis, which included five clinical trials with 264 young adult participants, found that virtual reality (VR) might be an effective tool for the management of acute postoperative pain in knee arthroplasty patients. This is consistent with a 2021 systematic review and adds to the growing body of evidence supporting VR’s utility in this context [25]. Although pain is studied in different articles, this review focuses specifically on acute postoperative pain after TKA; a type of pain little studied according to the results found. Nonetheless, caution is warranted because the quality of the evidence observed is low or very low. 

The primary outcome, pain intensity, showed statistically significant improvements, aligning with recent studies that emphasize VR’s role in distracting patients from acute pain [26,27]. Specifically, the results of the study, measured with the VAS scale, indicate that virtual reality is useful in relieving postoperative pain in the short term. Some research that also uses this measurement scale supports these results [28]. In addition, some studies even point out that VR could also be useful for the management of perioperative anxiety [29,30]. However, the quality of the evidence in these articles is rated as low or very low, and, therefore, more research is still necessary.

The key point in VR’s effectiveness is distraction. This may be effective due to the psychological component of pain processing, as explained by limited attention span theory and gate control theory [31]. This is because pain is not merely a physical response but also a psychological one [28]. This is why the negative emotions experienced by patients who have undergone surgery (fear, anxiety, etc.) may make them more susceptible to the perception of pain [28]. This relationship between the psychological response and pain can also be explained by physiology; when a patient experiences anxiety, sympathetic activity increases and endogenous adrenaline is produced, which, through the nociceptors, causes an increase in pain [32].

Several studies have demonstrated the efficacy of VR in other types of pain. In chronic pain, the results have been found to be clinically significant in terms of pain relief [33]. In perioperative pain, promising results in terms of perception have also been demonstrated [32]. Its efficacy has also been studied in other types of patients and in other clinical settings. For example, in the pediatric population, distraction with virtual reality has been effective in reducing pain [34], especially in needle-related procedural pain [35]. 

In our pooled analysis of samples, we found that virtual reality-based therapy improved functionality as measured by the WOMAC and HSS in the long and medium term, respectively, despite the evidence being of low quality due to the small sample size derived from the limited number of articles available for the meta-analysis. Only three studies assessed quality of life using the WOMAC scale [20,22,23].

These results are consistent with published data that demonstrated that virtual reality-based rehabilitation therapy, when compared to traditional rehabilitation, yielded better outcomes for WOMAC and HSS in the short and medium term [36]. However, some studies have provided opposing data, suggesting that this improvement in quality of life may be limited to the short term. Extended reality, which includes virtual reality, augmented reality, and mixed reality, was evaluated during postoperative rehabilitation in patients undergoing knee arthroplasty, comparing it with traditional rehabilitation. Extended reality showed an improvement in patients’ quality of life in the first month; however, it did not demonstrate an improvement in the following 6 months [29].

The improvement in the range of motion is one of the key aspects objectively assessed to determine the function of the knee [37]. However, no differences were found between the experimental group and the control group for the range of motion measured by short-term ROM. Our results contrast those of other authors, who reported a significant improvement in the experimental groups compared to the control between 2 weeks and 3 months after surgery [38,39]. 

Other previous studies were unable to meta-analyze this component due to high variability in terms of the methods and measurement units applied [36].

### 4.1. Limitations

This systematic and meta-analysis review has some limitations that should be considered when interpreting the results. One of the main limitations is the presence of a very limited number of studies, highlighting the need for further research on the topic. Only five articles addressed the use of virtual reality in the management of postoperative pain following knee arthroplasty [19,20,21,22,23]. It is important to note the low methodological quality of the available articles, as this compromises the reliability of the results. The ROB2 tool showed that all articles were classified as having a high level of risk, indicating the need for new clinical trials with stronger methodological rigor. Some articles did not contain sufficient information to be included in the meta-analysis. 

Furthermore, these articles presented numerous sources of heterogeneity that complicated the comparison process. Significant variability was observed among the studies regarding the applied VR protocol, as well as the duration and timing of application. Additionally, a large disparity was noted in the type of virtual reality program and device used in each study. All this variability complicated the process of obtaining meaningful data in the meta-analysis.

### 4.2. Clinical Implications

Virtual reality is expanding in clinical settings to support treatment and promote well-being [40]. At the treatment stage, it can be useful as a distraction method for pain management as a non-pharmacological treatment, to face uncomfortable medical procedures [41] and to reduce anxiety and the symptoms derived from it, since it enhances attention and helps the patient to feel more relaxed [30], thus promoting well-being and providing better quality care. Recent studies also demonstrate its usefulness in the field of rehabilitation [42].

In addition to acute pain, VR has also demonstrated its effectiveness in controlling chronic pain, for example, in different areas [43,44].

Moreover, recent studies support its effectiveness in other areas of health. In psychology, multiple studies have been carried out to reduce anxiety [45], establish exposure treatments for phobias [46] or treat depression [47].

It should be noted that the implementation of virtual reality in the hospital setting not only represents a significant technological advance, but is also emerges as an economically cost-effective therapy [48]. Previous studies have shown that it could decrease hospital costs by reducing the length of hospital stays. However, they did not find significant short-term differences between the savings from the reduction in opioid use and the expenses associated with virtual reality programs [49]. The use of virtual reality therapies has also been evaluated in comparison to mechanical therapies for pain management, revealing a substantial economic benefit [50].

The aforementioned clinical applications, added to the few adverse effects that it causes, the low cost and the easy accessibility of this technology, make VR an innovative technique with a wide range of applicability at the clinical level.

### 4.3. Future Lines of Research

The limitations identified in this systematic review highlight key points for the development of future research that can advance the understanding of the role of virtual reality in relieving postoperative pain. It is essential to enhance the quality of new studies by emphasizing the importance of designing a rigorous methodology to improve the reliability of the results. 

The standardization of virtual reality interventions is also presented as a crucial aspect to facilitate comparison between studies and enable the identification of more effective approaches. Additionally, it would be important to tailor the virtual reality experience to the specific preferences and needs of each individual, which could further enhance its therapeutic efficacy, as well as acceptance and adherence. 

Considering larger sample sizes and the diversity of populations, and considering factors such as gender or age, is essential to ensure the applicability and generalization of the results to broader clinical contexts. 

Despite virtual reality emerging as a potential non-pharmacological therapy that could contribute to the reduction of the use of opioids [51], none of the articles measured aspects related to the doses of the administered analgesics.

## 5. Conclusions

The evidence regarding the application of virtual reality in patients after total knee arthroplasty is very limited, despite experiencing considerable growth in recent years. Our findings suggest that the use of virtual reality during the postoperative period could be an effective non-pharmacological therapy in relieving acute pain, compared to a control intervention, with a very low degree of certainty. Additionally, the results also suggest that it may be effective in improving function/quality of life, with a very low and low degree of certainty, respectively. Finally, the results suggest that it would not have effects on the ROM compared to a control intervention, with a very low degree of certainty. Given the scarcity of available articles and the lack of methodological quality in existing ones, there is a clear need for new high-quality trials to confirm the effectiveness of virtual reality in relieving acute postoperative pain.

## Figures and Tables

**Table 1:** Characteristics and design of the included studies.

Author, Year, Country	Study Design	Patients Characteristics		Diagnosis	Intervention		Study Period	Outcome Measure	Follow-Up
		Participants (IG/CG); (Female) [Male]	Age (Years) (IG/CG)		IG	CG			
1 Lingfeng Li, 2022, China Ref. [19]	RCT	40 IG: 20; (12) [8] CG: 20; (10) [10]	IG: 33.6 ± 8.11 CG: 31.8 ± 7.36	Knee joint injury	IG: AR—based rhb training technology (real scene training data acquisition, virtual scene construction, and virtual and real fusion)	CG: Traditional rehabilitation (active and passive exercises)	During rehabilitation	VAS	6 weeks and 3 months after the surgery (HSS) 3 days, 7 days and 14 days postintervention (VAS)
2 Gianola et al., 2020, Italy Ref. [20]	RCTsimple blind	85 IG: 44 CG: 41 (48) [37]	68.6 ± 8.8	TKA	IG: VR rhb + passive knee motion on a Kinetec knee continuous passive motion system and functional exercises (stairs and level walking)	CG: Traditional rehabilitation (similar exercises for goals without the VR support system) + passive knee motion on a Kinetec knee continuous passive motion system and functional exercises (stair negotiation and level walking)	Daily for 60 min on at least 5 days	VAS, WOMAC, (EQ-5D), GPE score, frequency of medication intake	At baseline (3–4 days after TKA); at discharge (around 10 days after surgery)
3 S. Barry et al., 2022, EE.UU Ref. [21]	CT	18 THA IG: 8 CG: 16 TKA IG: 7 CG: 20 (67%)	CG: 74	THA and TKA	IG: Spinal anesthesia+ IVR (PICO G2 4K Enterprise goggles and Bose Quiet comfort QC 35 noise-canceling headsets, choice of the 4 different visual content environments created by HypnoVR and voice-guided relaxation technique sounds)	CG: spinal anesthesia	During the intervention	Intraoperative Quantity of sedation (preoperative and intraoperative); maximum heart rate; maximum systolic blood pressure; anesthesia time; OMEs Immediate postoperative outcomes PACU sedative/narcotic usage; vital signs; Numerical Pain Rating Scale; recovery duration	Preoperative, intraoperative, immediate postoperative and acute postoperative outcomes
4 Fuchs et al., 2022, Israel Ref. [22]	RCT	55 IG: 30 (19) [11] CG: 25 (13) [12]	Average age 70 years in the control and experimental group	Primary TKA	IG: Conventional physiotherapy + CPM equipment + Samsung Gear VR	CG: Conventional physiotherapy + CPM equipment	IG: CPM 15 min and Samsung Gear VR 15 min CG: CPM 15 min	VAS WOMAC	Before surgery, day 1 and day 2 post-operatively (VAS) Before surgery and six-months postoperatively (WOMAC)
5 Chi Jin et al., 2018, China Ref. [23]	RCT	66 IG: 33 (18) [15] CG: 33 (17) [16]	IG: 66.45 ± 3.49 CG: 66.30 ± 4.41	Osteoarthritis patients undergoing total knee arthroplasty	IG: VR rehabilitation (foot dorsiflexion and plantar flexion, exercises targeting quadriceps muscle strength and passive exercises on knee flexion with VR(Mide Technology Inc.)	CG: conventional rehabilitation(foot dorsiflexion and plantar flexion, exercises targeting quadriceps muscle strength and passive exercises on knee flexion)	Dorsiflexion and flexion: first day after TKA Quadriceps strength: from the second day after TKA IG: knee flexion with VR 30 min 3 times a day CG: knee flexion 3 daily sets of 30 repetitions	VAS WOMAC	1, 2, 3, 5 AND 7 days after TKA (VAS) Before TKA and 1, 3, 6 months after TKA (WOMAC)

IC: Intervention control, CG: Control group, RCT: Randomized control trial, CT: Control trial, RHB: rehabilitation, AR: Augmented reality, TKA: total knee arthroplasty, VAS: visual analogue scale, WOMAC: Western Ontario and McMaster Universities osteoarthritis index, EQ-5D: EuroQol five-dimension, GPE: global perceived effect, THA: Total hip arthroplasty, IVR: immersive virtual reality, CPM: continuous passive motion device, ROM: Range of motion, OMEs: intraoperative oral morphine equivalents.

**Table 2:** Summary table of virtual reality programs.

Author, Year	Hardware	Content (Scenario)	Interaction	Supervision Yes/No
Lingfeng Li, 2022 Ref. [19]	AR (unspecified)	Fusion scene of virtual and reality to guide the training. Initial stage: virtual knee joint in the exercise therapy Middle and late stage: aircraft roaming scene games	Training movements	Yes
Gianola et al., 2020 Ref. [20]	VR goggles	Games during rehabilitation	Training movements	Yes
S. Barry et al., 2022 Ref. [21]	PICO G2 4K Enterprise goggles, Bose Quiet Comfort QC 35 noise canceling headsets, choice of 4 different visual content environments created by HypnoVR and voice-guided relaxation techniques/sounds	Distract patients from their intraoperative environment by voice—guided relaxation techniques/sounds	No	Yes
Fuchs et al., 2022 Ref. [22]	Head mounted display that allows projection of a three-dimensional image (Samsung Gear VR)	The VR intervention included a movie that was chosen by the patient from several options, either a nature film or a music film. Patients underwent CPM physiotherapy for 15 min (one session per day) with VR headset.	Rehabilitation movements	Yes
Chi Jin et al., 2018 Ref. [23]	Virtual reality glasses	Patients were asked to row a boat using knee flexion (interaction of VR) in an immersive virtual environment for 30-min periods, three times a day	Rehabilitation movements	Yes

VR: virtual reality. AR: augmented reality.

**Table 3:** Certainty of evidence assessed with GRADE.

Certainty Assessment							No. of Patients		Effect		Certainty	Importance
No. of Studies	Study Design	Risk of Bias	Inconsistency	Indirectness	Imprecision	Other Considerations	Virtual Reality	Control	Relative (95% CI)	Absolute (95% CI)		
VAS pain (Scale from: 0 to 10 cm, decreasing values indicate improvement) (follow-up: range 2 days to 10 days)												
4	randomized trials	serious ^a^	serious ^b^	not serious	not serious	publication bias strongly suspected ^c^	118	122	-	MD 0.82 cm lower (1.3 lower to 0.35 lower)	⨁◯◯◯ Very low	CRITICAL
WOMAC (Scale from: 0 to 100 points, decreasing values indicate improvement) (follow-up: 6 months)												
2	randomized trials	serious ^a^	not serious ^d^	not serious	not serious	publication bias strongly suspected ^c^	63	58	-	MD 4.6 points lower (6.5 lower to 2.6 lower)	⨁⨁◯◯ Low	IMPORTANT
Hospital for Special Surgery Knee-Rating Scale (Scale from: 0 to 100 points, decreasing values indicate improvement) (follow-up: 3 months)												
2	randomized trials	serious ^a^	very serious ^e^	not serious	not serious	publication bias strongly suspected ^c^	53	53	-	MD 6.5 points higher (0.04 higher to 13 higher)	⨁◯◯◯ Very low	IMPORTANT
Range of Motion (Grades, increasing values indicate improvement) (follow-up: 7 days)												
2	randomized trials	serious ^a^	very serious ^f^	not serious	not serious	publication bias strongly suspected ^c^	68	72	-	MD 3.4 grades higher (6 lower to 12.8 higher)	⨁◯◯◯ Very low	IMPORTANT

CI: confidence interval; MD: mean difference, ^a^. High overall risk of bias of the included studies, ^b^. Moderate heterogeneity I2 = 70%, ^c^. Egger’s test reached statistical significance, ^d^. Low heterogeneity I2 = 13%, ^e^. High heterogeneity I2 = 93%, ^f^. High heterogeneity I2 = 87%.

## Data Availability

The relevant data are available upon request to the corresponding author.
